# Role of kinetic chain in sports performance and injury risk: a narrative review

**DOI:** 10.25122/jml-2023-0087

**Published:** 2023-11

**Authors:** Haifa Saleh Almansoof, Shibili Nuhmani, Qassim Muaidi

**Affiliations:** 1 Department of Physical Therapy, College of Applied Medical Sciences, Imam Abdulrahman Bin Faisal University, Dammam, Kingdom of Saudi Arabia

**Keywords:** eccentric strength, neurocognitive control, muscle activation, injury risk

## Abstract

The kinetic chain refers to the body's intricate coordination of various segments to perform a specific activity involving precise positioning, timing, and speed. This process is based on task-oriented and activity-specific pre-programmed muscle activation patterns enhanced by repeated practice. It demands muscular eccentric strength, joint flexibility, and musculotendinous elastic energy storage. The body core (lumbopelvic–hip complex) forms the kinetic chains’ central point of activities in most sports because it facilitates load transfers to and from the limbs. The kinetic chain relationship with fascia, peripheral nerves, and tensegrity is fundamental to holistic human body movements. The kinetic chain function demands neuromuscular, sensorimotor, and neurocognitive control. Any blockage or defect in the kinetic chain can develop compensatory patterns, high demands on distal parts, and overuse and overload injuries. Taking a holistic approach and evaluating the integrity of the kinetic chain in athletes can significantly enhance efforts to improve sports performance and mitigate injury risk.

## INTRODUCTION

The interconnected musculoskeletal kinetic chain is a complex motor unit and the base of human movements [1, 2]. The kinetic chain is the sequenced and coordinated activation of body segments that puts the terminal/distal part at the optimal timing in the optimal position with the optimal speed to perform the required athletic activity [3].

The human body's ability to perform complex tasks relies on the intricate coordination of various dynamic systems, the neuromuscular and sensorimotor control systems being essential for this task [4]. These systems are responsible for processing environmental input, employing feedforward [5, 6] and feedback loops [7]. The kinetic chain energy transfer across the body is a mechanism that engages the neuromuscular coordination of the body’s segments, allowing for sequential movements [8]. The contribution of more body segments in the total force output production leads to a higher potential velocity at the distal part [8]. Therefore, when the muscle chains are used in ways that disturb the parallel agonist/antagonist co-contraction, the highly sensory muscle chains can disrupt movements.

The neurocognitive function integrates and processes visual focus, self-monitoring, agility, dual-tasking, accurate motor performance, reaction time, and speed. In the context of collegiate female athletes, lower neurocognitive function has been associated with a shift toward dominant muscle activity patterns in the quadriceps [9]. This alteration in kinetic chain muscle activities can increase the risk of anterior cruciate ligament (ACL) injuries [10].

### Kinetic chain, fascia, and neural tissue

The fascia is a fibrous connective tissue that forms an extensive web-like network throughout the body, supporting the spine and facilitating the transfer of loads between the core and limbs [11]. This interconnected network of fascial tissues significantly influences the biomechanics of the body. Research has shown a positive, strong, and significant correlation between the density of myofibroblasts and contractile response in these tissues. This suggests that myofascial tissue tension is actively regulated by myofibroblasts and has the potential to impact the dynamic functioning of the musculoskeletal system. The influence of myofascial tissues is high enough to potentially affect motoneuronal coordination. Myofascial chains explain why muscles in the human body do not function as isolated units but instead operate in an interconnected manner, forming a systemic and continuous network [12]. A systematic review concluded that several muscular myofascial chains are anatomically strongly evidenced [12]. These integrated myofascial connections among muscles play a significant role in maintaining the stability of the human skeleton [13, 14].

Muscles tend to work synergistically, functioning as bigger anatomical interlinked chains [15]. Myofascial chains are distinct muscle groups united by the fascial system [12]. The superficial back line (one of the myofascial chains) involves the plantar fascia, Achilles tendon, triceps surae, hamstrings, sacrotuberous ligament, and erector spinae [16] that can facilitate effective force transmission between the core and limbs through its anatomical integration [17]. The thoracolumbar fascia connects the lower limbs (through its attachment with the gluteus maximus) and the upper limbs (through its attachment with the latissimus dorsi) [18], permitting the core to help in coordinated kinetic chain movements, such as throwing [19]. The thoracolumbar fascia attaches to the transverse abdominus and internal oblique muscles, offering the lumbar spine three-dimensional support and core stability [19]. It forms a stabilizing ‘ring’ surrounding the abdomen, made of the thoracolumbar fascia, the abdominal fascia, and the oblique muscles, acting like a stabilizing corset [20]. In addition, the thoracolumbar fascia channels load transmissions between the limbs and core [21].

In the lower limb, the long head of the biceps femoris is in continuity with the sacrotuberous ligament, which in turn is connected to the thoracolumbar fascia [11]. Research has shown that when the biceps femoris tendon is pulled laterally, it can displace the interspinous ligament between the fifth lumbar vertebra and the first sacral vertebra, highlighting the load-transferring role of these fascial connections even between distant body areas and joints [11]. A dissection study has revealed various continuous fascial connections between the pelvis and the feet, including the iliotibial band, femoral intermuscular septa, crural fascia, and crural intermuscular septa [22]. These fascial connections can transfer loads between the pelvis and the feet [23]. Passive neural tissue can also influence the transfer of load between the pelvis and the lower limb [23]. For instance, higher tension in the sciatic and tibial nerves was observed when hip flexion and ankle dorsiflexion were combined [24]. Similarly, the combination of hip flexion with the knee in an extended position (i.e., long sitting) led to the greatest reduction in ankle dorsiflexion [23]. Moreover, knee joint range of motion (ROM) is reduced when hip flexion and ankle dorsiflexion are combined [25]. Since no single muscular structure passes the lower limb joints, non-muscular structures (i.e., fascia and neural tissues) can alter/control forces working on distal joint mechanics [23]. In a sitting position with trunk rotation, significant differences in ankle dorsiflexion ROM can occur due to the tensile force generated by trunk rotation being transmitted to the contralateral distal end, thereby altering ankle dorsiflexion ROM [26]. This transmission or propagation of tensile force is facilitated through the myofascial chain and the posterior oblique sling, particularly the one connecting the trunk with the contralateral triceps surae [26].

The concept of integrated kinetic chain asserts that muscular chains/pathways are interlinked through soft tissue viscoelastic envelopment of polyarticular myofascial chains. The myofascial chains can transfer force, provide sensory and neuromotor input, and act like organized muscle synergies. The viscoelastic myofascial chains work within the model of bio-tensegrity, necessitating eccentric function, end-range motions, joint stability, and elastic energy storage. In a study published in 2017, the authors utilized essential myofascial chain/pathway concepts to provide a comprehensive illustration of how ROM measurements at a single joint (specifically, the hip joint) depend on the positioning of the entire body in postures mimicking those encountered during sports activities, particularly those related to football [27].

Repetitive movements, a common requirement in nearly all sports, can impact the fascia surrounding overused muscles, causing it to shorten and thicken while elongating in other areas [28]. Muscles execute movements in kinetic chains, although the muscle function is not usually tested in its kinetic chain [29, 30]. Isolated tests do not examine the movement patterns related to kinetic chains [31]. The full functionality of fascia in a specific chain is manifested by permitting all the muscles to activate and hold the body in the chain test position [31]. Myofascial chain restrictions are manifested as the inability to hold the position and/or discomfort in keeping the position [31].

### Kinetic chain and bio-tensegrity

Human movement is multisystemic and complex [1]. Understanding how these systems interact in human movement can enhance our understanding of injury causes, prevention, and rehabilitation [1]. This perspective views human movement as a holistic, interconnected, complex system rooted in bio-tensegrity [1]. Bio-tensegrity is a concept where the bones are joined/linked with multiple viscoelastic myofascial chains with muscle tone maintained in a tension-dependent manner [1]. The tensegrity concept can explain how the human kinetic chain is interconnected and interdependent [13, 17]. Tensegrity concepts regard the musculoskeletal system as a series of elements that resist compression (i.e., the bones) and are interconnected by a continuous network of viscoelastic elements (i.e., the musculotendinous system), which provides constant elastic tension within the system, both at rest and during movement [13, 32, 33]. The design of the bio-tensegrity system is evident in the continuous adjustments made by the entire musculoskeletal system, creating global patterns during movements [1]. The viscoelastic myofascial muscle chains function within a bio-tensegrity design that emphasizes the importance of addressing human movement holistically [1]. The influence of whole-body positioning on the range of motion of a single joint illustrates the kinetic chain's function through the myofascial muscle chain [1].

### The kinetic chain and the core

The core (lumbopelvic–hip complex) forms the kinetic chains’ central point of activities in most sports and is essential in injury risk mitigation [18]. Core stability is the capability to control the trunk alignment and movement over the pelvis and lower limbs to permit optimal force and motion generation, transfer, and control to the distal part in an integrated kinetic chain [18]. Therefore, core stability is crucial to enhance athletic function efficiency by maximizing the function of the upper and lower limbs’ kinetic chains.

The kinetic chain involves the sequential activation of muscles while performing a specific task, relying on pre-programmed patterns of muscle activation that are enhanced by repetition [18]. Muscle activation patterns associated with fast upper-limb movement reveal that the contralateral gastrocnemius/soleus are activated first [34], and then the activation reaches up (through the core) to the arm [35]. In baseball throwing, the muscle activation for pitching starts from the contralateral external oblique and continues up to the upper limb [35].

The trunk/core muscle activation patterns enhance the muscle activation patterns of the limbs in both stability and mobility, while the distal muscle activity tends to be more specialized for precision tasks [18]. Core muscle activation is pivotal in generating rotational torques around the spine, typically initiating on the contralateral side to produce force and rotational movement [35, 36]. Furthermore, core muscle activation provides stiffness to the torso, forming a base against which limb musculature can be stabilized while contracting [20, 36].

Core proximal activation is essential for facilitating coordinated movements of distal segments [18]. Core proximal activation provides the precision and stability for the whip-cracking-like upper extremity distal segment maximal force (e.g., when throwing a ball, the core muscles stabilize the trunk to give the needed base for the arm to throw the ball with force and precision). The upper extremity distal part is smaller than the proximal part and, therefore, has a minimal moment of inertia, which allows higher velocity summation. Consequently, the combination of core activation and hand minimal moment of inertia can allow the ball to be thrown with high precision, power, and acceleration [18]. The core also helps control force. In kicking, maximum force at the foot is generated by the propagated moment after the hip joint flexion [3]. The periscapular and core muscle activation represents almost 85% of the muscle activation needed to control the forward-moving upper limb [37]. It was found that tennis players with lower knee flexion ROM during the back-swing phase of the serve action had 23–27% greater shoulder rotation, horizontal adduction stress, and elbow valgus stress [38]. The possible explanation was that the lower knee flexion ROM caused breakage in the kinetic chain and less contribution from the hip and core [38].

### Kinetic chain and sports performance

Sports performance depends on relevant kinematic and kinetical variables [39]. Sports performance improvement is correlated with injury prevention [39]. The kinetic chain refers to the sequential activation of task-specific body segments, enabling efficient generation, summation, and transfer of mechanical energy to support functional movement patterns [40, 41]. Kinetic chain inefficiency occurs when there is a defect or disruption at any point within the chain, which affects the transfer of energy or force to nearby segments [40, 41]. The defect in the kinetic chain places additional demands on the remaining segments of the chain to compensate for the energy loss [40]. This compensation has been identified as a contributing factor that increases the risk of shoulder pain and injury during overhead sports activities [40, 42]. In the dominant tasks of the upper extremity, the energy generation and production are in a proximal-to-distal sequenced pattern [43]. For example, during a tennis serve, approximately 50%–55% of the total required kinetic energy is generated from the legs and trunk [41, 44]. In elite handball players, the main determinant of the throwing velocity is the lower-limb peak power [45]. Moreover, lower limb peak power was strongly correlated with the sprint-swim speed in freestyle swimmers [46]. Furthermore, lower limb musculature mass and contraction velocity correlate significantly with the performance of javelin throwing [47].

The muscle activation sequence in striking and throwing skills follows a proximal-to-distal direction. The "tension arc" travels along the body from the contralateral arm on the non-kicking side to the kicking leg as it extends and abducts, resulting in trunk rotation [48]. The forward movement of the kicking limb and the contralateral arm releases this tension arc, allowing it to shorten and demonstrate the stretch-shortening cycle [48]. For kicking to happen, the trunk generates a sequential proximal-to-distal energy flow from the trunk to the lower limb to execute angular motions to contact the ball. The strategies involved in whole-body energy transfer during instep kicking include energy absorption by the support limb, the formation of an eccentric 'tension arc' between the torso and kicking hip, and its concentric release, along with proximal-to-distal sequencing of the kicking limb during the downswing [49]. In maximal instep soccer kicking, skilled athletes exhibited greater trunk rotation range of motion and speed, resulting in higher ball velocity compared to novice athletes [50].

For baseball pitching to occur, a complex muscle activation sequence along the kinetic chain generates and efficiently transfers the required energy for executing a baseball throw [51]. The kinetic chain influences the activation of the scapular musculature (serratus anterior) throughout the practice of knee push-up-plus exercises. Electromyography studies have demonstrated that ipsilateral lower-limb extension amplifies the activation of the serratus anterior, whereas contralateral lower-limb extension reduces its activation [52]. Consciously contracting the abdominal muscles was an effective strategy to magnify the serratus anterior and lower trapezius activity (as proven by the electromyographical readings) during the push-up plus phase [53].

Efficient energy transfers involve the sequential transfer of energy from the larger and more proximal parts of the body to the smaller terminal parts [49]. Efficient energy transfers along the kinetic chain were linked to a lower risk of injury and higher performance [49] because a synchronized kinetic chain can reduce joint loads, enhance velocity, and increase force production throughout the motion [54]. Higher-level players use shoulder and wrist power better by effectively engaging the entire body kinetic chain [55]. This power generation process during throwing starts in the lower limbs and core, where large and powerful muscles are located [56]. Around half of the force production throughout the throwing motion occurs in the hip and trunk [51]. The lower-limb-generated force is transferred through the trunk to the scapula of the throwing/accelerating arm [51]. Mobility restriction and kinematics alteration in the hip and trunk are linked to a throwing mechanics breakdown (loss of energy production) and shoulder and elbow injuries [57, 58]. The mobility restriction causes a loss of energy production and consequent larger force production role on the arm, placing abnormal and damaging stresses on the soft tissues [51, 59, 60].

### Kinetic chain and sports injuries

Sports injuries are complex phenomena due to the interplay of various risk factors or predictors (known as the risk profile) that can lead to compensatory patterns and eventual injuries [7]. The ability to predict and prevent sports injury depends on identifying these risk patterns (risk profile) through a non-linear and complex system approach [7]. In the kinetic chain, disruptions at proximal links can increase demands on more distal segments, requiring enhanced functional abilities in those areas and making them more susceptible to injuries [39, 61, 62].

Deficits in the kinetic chain links in the trunk and lower limbs were present in approximately 50% of cases involving injuries to the superior glenohumeral labrum anterior and posterior regions in throwing shoulders [57]. In individuals with chronic ankle instability, changes in ankle-joint kinetics, including a decrease in ankle-eversion moment and an increase in ankle plantar-flexion moment, were observed during side-cutting task performance. Additionally, increased hip joint stiffness was observed [63]. Such altered lower-limb kinetics and movement patterns may increase the risk of recurrent lateral ankle sprains [63]. Soccer players were eight times more prone to hamstring strain injury if their hamstring muscles were activated after the lumbar erector spinae (normally, the hamstring is to be activated before the erector spinae) during prone hip extension (mid-range and end-range) [64].

The kinetic chain concept implies that the disorder of a joint can precipitate injuries to other joints (usually distal to the joint involved) [65]. Athletes who are landing with altered lower-limb mechanics (with dynamic knee valgus, tibia internal rotation, and pronated feet) have a high risk of sustaining acute ACL non-contact injury [66] and anterior knee pain as overuse injury [65, 67, 68]. In alpine skiers, tibia internal rotation with a knee full extension or flexion beyond 90° was linked to a non-contact ACL injury [66]. More proximally, impaired trunk control, motion, and body-weight shift on the lower limb were linked to a high risk of ACL non-contact injuries [66].

### Kinetic chain-related clinical tests

#### The closed kinetic chain upper extremity stability test (CKCUES test)/ Davis test

The closed kinetic chain upper extremity stability test (CKCUES test) is a valuable, cost-effective clinical tool for evaluating shoulder performance [69]. It provides quantitative data for assessing upper extremity function in a closed kinetic chain context [54]. This test targets explicitly the stability of the scapular muscles, making it useful for evaluating scapular stability [70]. Furthermore, it determines the upper limb function, progression in rehabilitation, and return-to-sport judgment [71]. The CKCUES test shows strong reliability and validity [71] and is designed to be user-friendly for clinicians, making it easy to administer and comprehend [72]. The test score is determined by counting the number of times the individual, while in a plank position, is touched by their swinging hand and supporting hand [72]. Its performance strongly correlates with the isokinetic strength of shoulder flexors and elbow extensors at 180°/s for men [71]. It is strongly associated with the isometric strength of serratus anterior and moderately with that of triceps brachii [73].

#### The Bunkie test

The Bunkie test examines kinetic chains across the fascia and detects the apparent restrictions in the kinetic chains along the fascia lines [31]. Mayer's anatomy trains serve as the foundation for the kinetic chains employed in this test [29]. Each kinetic chain is associated with a particular testing position, with both the right and left sides of the body being evaluated. Participants are required to maintain the testing position for 40 seconds. There is a significant correlation between performance constructs (agility, speed, explosive power, and muscle endurance) and performance in the Bunkie test in healthy rugby players [31].

#### Closed kinetic chain dynamic lower extremity stability (CKCLE) (developed by Lee, Hwang, Jung, Ahn, and Kwon, 2020)

The closed kinetic chain dynamic lower extremity stability (CKCLE) test is a novel test examining the functional performance and assessing antigravity posterolateral hip musculature function. During this test, individuals are instructed to lift one foot, touch it to the opposite knee, and then lower it back to the floor while isometrically maintaining the bridging position. This motion is repeated for 20 seconds, and the score is based on the number of touches achieved in that time frame. There is a positive and significant correlation between the strength of the supporting lower-limb hip extensors, abductors, and external rotators and the number of foot touches completed in 20 seconds [74].

#### Closed kinetic chain lower extremity stability test (CKCLEST) (developed by Arikan, Maras, Akaras, Citaker, and Kafa, 2021)

The closed kinetic chain lower extremity stability test (CKCLEST) is a novel, easy, and cost-effective performance-based comprehensive test developed to examine the lower limb endurance, strength, power, trunk, whole-body stability, functional lower limb stability, core, lower limb static/dynamic control, and quadriceps and hamstring muscles coactivation at the same time. CKCLEST is an objective measurement tool that is valid and reliable. CKCLEST evaluates the individual’s function of the closed kinetic chain. CKCLEST is not a plyometric test and does not have compressive forces on joints [75]. CKCLEST has excellent test-retest, intra-, and inter-rater ICC values ranging between 0.83 and 0.93 [75]. During the CKCLEST, the individual begins in a forearm plank position with feet shoulder-width apart and toes touching the floor, maintaining a straight and isometric body position. They are then instructed to alternate crossing one foot over the outer side of the contralateral foot and returning it to the starting position, followed by the same movement with the other foot. This alternating movement between the feet is performed as rapidly as possible. The score for the test is determined by the number of repetitions completed in a 15-second time frame, with both average and best scores being considered [75].

**Figure 1:**
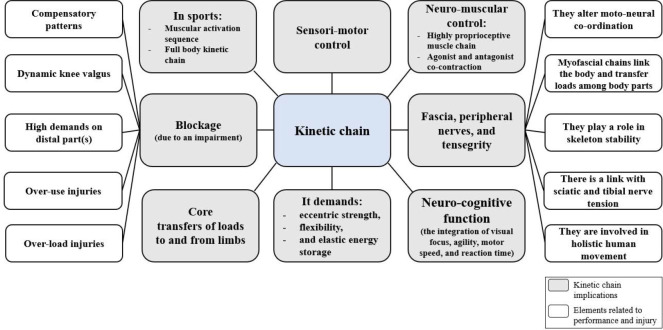
Summary of kinetic chain implications and elements related to injury and sports performance

In summary, the kinetic chain and its links with the nervous system and myofascia make the base for understanding the interdependence of various body parts in performing any activity. This interdependence has significant implications for sports performance and the risk of injury, as illustrated in Figure 1.

## CONCLUSION

The kinetic chain is the basis of sequenced and coordinated human movements facilitated by the nervous system and myofascia and explains the interdependency between body parts. Myofascial chains and passive neural tissue dynamics facilitate effective force transmission between the core and limbs. A single joint ROM is governed by the whole-body positioning that echoes the kinetic chain work through the myofascial muscle chain. Sports-related repetitive movements lead to the thickening and shortening of the fascia around the overused muscles and fascia lengthening elsewhere. The core is the central point of the kinetic chains in most sports, and therefore, it is essential for decreasing the risk of injury and maximizing the limbs’ function. The intricate sequence and coordination of muscle activation within the kinetic chain are essential for efficiently transferring the required energy to execute sport-specific skills or tasks. Any defect within the kinetic chain places demands on the other parts to adjust for the energy loss, eventually raising the risk of sustaining injuries in sports. Tests related to the kinetic chain provide quantitative data by engaging the core isometrically and the limbs dynamically. Therefore, it is reasonable for those responsible for athlete health and sports performance to evaluate kinetic chain function, identify any issues, and address them to enhance performance and prevent injuries.
